# Comparison of molecular methods for *Bartonella henselae* detection in blood donors

**DOI:** 10.1371/journal.pntd.0011336

**Published:** 2023-06-01

**Authors:** Marina Rovani Drummond, Luciene Silva dos Santos, Amanda Roberta de Almeida, Karina de Almeida Lins, Maria Lourdes Barjas-Castro, Pedro Paulo Vissotto de Paiva Diniz, Paulo Eduardo Neves Ferreira Velho

**Affiliations:** 1 Applied Research in Dermatology and Bartonella Infection Laboratory, University of Campinas—UNICAMP; Campinas, Sao Paulo, Brazil; 2 Hematology and Hemotherapy Center, University of Campinas–UNICAMP, Campinas, Sao Paulo, Brazil; 3 College of Veterinary Medicine, Western University of Health Sciences, Pomona, California, United States of America; 4 Division of Dermatology, Department of Medicine, UNICAMP, Campinas, Sao Paulo, Brazil; Laos-Oxford-Mahosot Hospital Wellcome Trust Research Unit, LAO PEOPLE’S DEMOCRATIC REPUBLIC

## Abstract

The *Bartonella* genus consists of neglected pathogens associated with potentially transfusional-transmitted and fatal human diseases. We aimed to evaluate *Bartonella* sp. prevalence in 500 blood donors and compare the results with the data already published about these samples. We used molecular diagnostic methods to detect *Bartonella* sp.-DNA from blood and liquid culture samples: (A) conventional PCR for two gene regions, the ITS targeting the genus *Bartonella* and the specific *gltA Bartonella henselae*; (B) nested PCR for the *ftsZ* gene and (C) qualitative real-time PCR for the *gltA* gene, both *B*. *henselae* specific. We obtained 30/500 (6%) DNA detections from the blood samples; 77/500 (15.4%) DNA detections from liquid culture samples and five (1%) samples had DNA detection from both. In total, we detected *B*. *henselae* DNA from 102/500 (20.4%) donors. The samples used in this study had already been submitted for *Bartonella* sp.-DNA detection using only a conventional PCR in liquid culture. Sixteen samples (3.2%) were positive previously, and from these 16 samples, 13 were negative in the new investigation. We concluded that the use of liquid culture combined with different molecular tests increases the possibility of detecting *Bartonella* sp.-DNA, but the tests do not avoid false-negative results. More than a fifth of blood donors had at least one PCR that detected *Bartonella* sp.-DNA among the eight molecular reactions performed now (four reactions in whole blood and four in liquid culture). Seven percent had *B*. *henselae*-DNA detection for two or more distinct regions. Considering the results obtained previously, the DNA of *Bartonella* spp. was detected or the agent isolated in 23% of analyzed blood donors. The results establish that the low bacteremia and the fastidious characteristics of the bacterium are challenges to laboratory diagnosis and can make it difficult to confirm the infection in patients with bartonelloses.

## Author summary

Bartonella are bacteria that can infect humans and cause fatal diseases. They can cause chronic infection and can potentially be transmitted by transfusion since they infect red blood cells. They are difficult to cultivate in a laboratory, and they are present in low numbers in blood. There is no laboratory test that is sufficiently sensitive to detect them. To compare several laboratory diagnostic tests, we searched for these bacterial DNAs in blood and in microbiological liquid cultures of 500 blood donors. We used diverse molecular techniques and then compared the results with the previously published project. We concluded that the use of liquid culture combined with different molecular tests increases the possibility of detecting *Bartonella* sp.-DNA, but the tests do not avoid false-negative results. We found *Bartonella henselae* DNA in the blood of at least one in five donors. Hemovigilance programs are unlikely to contribute substantially to the identification of chronic posttransfusion infections since they are designed to identify well-defined acute outcomes, so it is urgent to review *B*. *henselae* transfusional risk of transmission and *Bartonella* sp. infection diagnosis in donors and in patients.

## Introduction

The genus *Bartonella* comprises small coccobacillary, gram-negative, facultative intracellular bacteria belonging to the alpha-2 subgroup of the phylum Proteobacteria [1]. One of their most important characteristics is fastidious growth [2–5]. They infect erythrocytes and endothelial cells, usually causing chronic and cyclic bacteremia in their hosts [4,6–8]. Bacteria of this genus have been linked with many diseases, one of them described from pre-Inca times [9,10].

The genus *Bartonella* has more than 47 species and subspecies, and at least 17 of them have already been related to clinical manifestations in humans [5,7]. Of these, three species are associated with the largest number of diseases: *Bartonella bacilliformis*, *Bartonella quintana* and *Bartonella henselae* [11], the latter being the most frequent species [12–14].

Several manifestations have already been related to infection by *Bartonella* spp. including Carrión disease (*B*. *bacilliformis*) [15]; trench fever, culture-negative endocarditis, bacillary angiomatosis, and chronic bacteremia (*B*. *quintana*) [14,16]; cat scratch disease (CSD), ocular manifestations such as Parinaud’s syndrome and neuroretinitis, bacillary angiomatosis, culture-negative endocarditis, bacillary peliosis, fever of undetermined origin, encephalopathy or osteomyelitis (*B*. *henselae*) [14,17,18]. Other manifestations, such as malaise, fatigue, insomnia, memory loss, splenomegaly, hepatitis, and meningitis, have also been described, and in some cases, the infection can be fatal [14,15,19–23].

Blood-sucking arthropods are the main mode of *Bartonella* transmission [6,24,25]. Other types of transmission, such as percutaneous accidents, transplantation of solid organs (kidney and liver), and vertical transmission, have been linked with infections by *Bartonella* spp. [22,26–29]. Transmission by blood transfusion is also possible. In a case reported by Pons *et al.*, transmission of *B*. *bacilliformis* was described after platelet transfusion from an asymptomatic blood donor [30]. A study conducted at UNICAMP showed that *B*. *henselae* remained viable for 35 days in experimentally infected blood bags stored at 4°C [31]. This study was used by the American Association of Blood Banks (AABB) to include the bacterium as a pathogen that can be transmitted by blood transfusion. In another study, Ruiz *et al*. demonstrated that *B*. *bacilliformis* remained viable in samples collected from patients with symptoms of Carrión disease after 30 months of storage at 4°C [32]. A study by Silva *et al*. detected the DNA of *B*. *henselae* in the spleen of mice that received transfusion of blood from animals that had been experimentally infected, although molecular blood tests were negative in all transfused animals [33].

Asymptomatic blood donors infected by *Bartonella* spp. may carry this bacterium in his red blood cells, posing a real risk of infection to blood recipients. However, laboratory confirmation of *Bartonella* sp. infection remains a major challenge. The fastidious nature of this genus, even in a specific culture medium, limits the diagnostic use of blood or tissue cultures [4,8,14,34]. Molecular methods have expanded the detection of *Bartonella* sp.; however, no current diagnostic method is able to confirm infection by *Bartonella* spp. in all infected patients, since these bacteria have low bacteremia, which makes detection even more difficult [35]. In addition, different molecular methods may yield distinct results. A study conducted with cats observed that 27.7% (31/112) of the animals had *B*. *henselae*-DNA detected in conventional PCR tests performed in liquid cultures. The same material was examined with nested PCR, and 45.5% (51/112) of all cats had DNA of *B*. *henselae* detected. DNA extracted directly from blood was also tested by nested PCR, and *B*. *henselae* DNA was detected in 76.8% (86/112). If we consider all tests performed with blood and liquid culture samples, 90.2% (101/112) of cats had *B*. *henselae*-DNA detected [36]. Therefore, a combination of several PCRs from different regions and from different samples increases the chances of detecting the pathogen.

A published study investigated the prevalence of *Bartonella* species in a population of blood donors using just one conventional blood liquid culture PCR. Blood samples from 500 voluntary blood donors were incubated in BAPGM (*Bartonella* alpha-proteobacteria growth medium) liquid medium and cultured at 37°C in 5% CO_2_ for 14 days. Then, the samples were subinoculated on agar medium containing 30% sheep blood for another 42 days. DNA was extracted from the liquid culture and tested by *Bartonella* sp.*-*specific conventional PCR, which amplifies the ITS region. The amplified products were sequenced to identify the species. Gram-negative isolates obtained from solid culture were also tested by the same technique. Sixteen blood donors (3.2%) were positive for *Bartonella* spp. in PCR after culture in liquid and solid media. DNA sequencing confirmed the homology of 15 samples with *B*. *henselae* and one sample with *Bartonella clarridgeiae* [37,38].

The present study aimed to evaluate the presence of *Bartonella*-DNA in blood and liquid culture of 500 blood donor samples from the University of Campinas (UNICAMP) Blood Bank, Campinas, Sao Paulo, Brazil, using four different PCRs in whole blood and in liquid culture samples and compare the results obtained in the previous project and already published [37], since the samples were the same.

## Methods

### Ethics statement

This project was submitted to the University of Campinas Institutional Review Board (IRB) under n°122/2005, and formal written consent was obtained from donors who agreed to participate in the research. In 2015, the IRB reapproved it under n°1.135.941 for further tests.

### Samples

This study analyzed two samples (whole blood and liquid culture) of each 500 blood donors from the University of Campinas (UNICAMP) Blood Bank, randomly collected from November 2009 to January 2010 during a blood donation procedure. These were the same samples used in a previous study that has already been published [37]. Epidemiological data about blood donors, such as gender; occupational animal exposure; contact with cats, dogs, other companion animals, bites from dogs, cats, and other animals; arthropod bites caused by ticks, fleas, or other insects; previous blood transfusion, etc., were already analyzed and published [39].

Whole blood samples collected in tubes with EDTA and liquid culture (another aliquot of whole blood incubated in BAPGM liquid medium and cultured at 37°C in 5% CO_2_ for 14 days) were stored at -20°C.

### DNA extraction

DNA extraction from whole blood and from liquid culture was performed using a QIAamp DNA Mini Kit (Qiagen). Controls were added to each extraction following the protocol already described [37].

### PCR

Controls were used in each reaction, and molecular techniques were performed carefully to avoid contamination following the procedures described in previous work [37].

All samples (both DNA extracted from the whole blood and liquid culture) were tested for all PCR techniques described below. Promega enzyme (GoTaq Flexi) was used in all reactions, except for qualitative real-time PCR. The PCR primers and conditions are described in Table A in S1 Appendix.

### Quality control PCR

The quality of the extracted DNA and the absence of PCR inhibitors in DNA samples were tested by the amplification of a fragment of the *GAPDH* (glyceraldehyde-3-phosphate dehydrogenase) gene, which encodes a glycolysis enzyme expressed by all mammalian cells [40].

### Conventional PCR

Two different reactions for the target gene were performed: the ITS region, or the 16S-23S rRNA intergenic region, for *Bartonella* spp. [41]; and the *gltA* gene, or the citrate synthase gene, for *B*. *henselae* [42].

### Nested PCR

A species-specific nested PCR was used in this study for the target region that encodes protein FtsZ that plays a role in cell division of *B*. *henselae* [43].

All PCR products were submitted to electrophoresis in 1.5% agarose gel stained with GelRed and visualized in a photodocumenter with UV light.

### Qualitative real-time PCR

The samples were tested by real-time PCR using the same primers used in conventional PCR for amplification of the citrate synthase gene (*gltA*) in the Sybr Green system using enzyme Fast SYBR Green Master Mix (ThermoFisher Scientific) [42]. In this study, real-time PCR results were used as qualitative PCR, considering results as positive or negative. In addition to the amplification curve, the melt curve was analyzed in comparison with the curve of the diluted *Bartonella* DNA used as a positive control. We considered positive samples with melt curves between 73.08°C and 73.41°C. We also performed electrophoresis, and the positive samples were confirmed by the presence of a band in the 1.5% agarose gel stained with GelRed.

### Sequencing

Amplified DNA with enough concentration was sent for Sanger sequencing. The results were analyzed using Chromas 2.6.6 software and compared to the GenBank database using the BLAST tool from the National Center for Biotechnology Information (NCBI).

### Statistical analysis

The McNemar-Bowker test was performed to decide which type of sample (blood or liquid culture) would be the most appropriate considering the results of any PCR method. This test is an extension of the McNemar test when there are nominal variables with more than two categories of nonindependent variables.

Bayesian latent class model (LCM) analysis was performed to find the best species-specific PCR method for *B*. *henselae*, regardless of the tested sample, and the best PCR method for blood or liquid culture samples. This test assumes that none of the tests is perfectly accurate; then, an ‘imperfect gold standard’ model is defined according to the results of multiple diagnostic tests with the same samples. The Bayesian approach can infer the prevalence of the studied agent and the test properties by adjusting the possibility of conditional dependence between the tests [44]. The limiting factor of Bayesian LCM analysis refers to the need for intense computer programming. In our study, an online tool was used (*Modeling of Infectious Disease Centre–Imperfect Gold Standard Model*) with results from three different PCR methods analyzing the same samples. The number of repetitions of the analysis was set at 25,000. Accuracy measures such as sensitivity, specificity, positive predictive value, and negative predictive value [45] were calculated using this method, as well as the 95% confidence interval. All *p* values were considered statistically significant if *p* < 0.05.

Additionally, the receiver operating characteristic (ROC) curve method was applied. This analysis uses a simple graphic method to study the variation in sensitivity and specificity for different cutoff values. The area under the ROC curve (AUC) is associated with the discriminatory power of a diagnostic test [46].

## Results

### PCR

All participants had whole blood and liquid culture analyzed, and all extracted samples had amplified in the quality control PCR (GAPDH), demonstrating the presence of DNA and the absence of PCR inhibitors.

**Fig 1:**
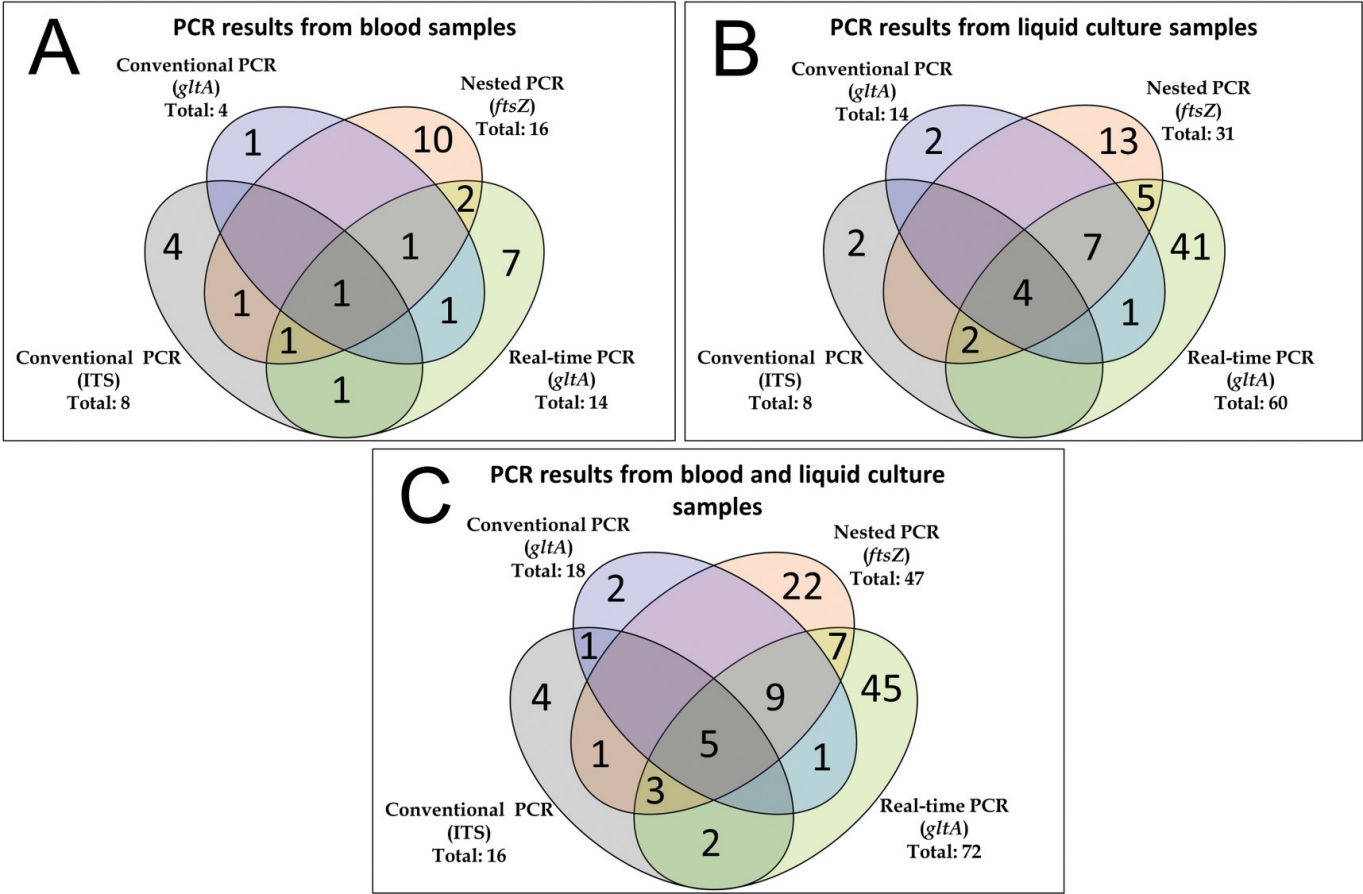
*Bartonella* sp.-PCR results from blood donors represented as Venn diagrams, showing how many samples were amplified in each reaction and detected in more than one PCR. **A:** *Bartonella* sp.-DNA was amplified in 30/500 (6%) blood samples. **B:** *Bartonella* sp.-DNA was detected in 77/500 (15.4%) liquid cultures. **C**: *Bartonella* sp.-DNA was detected in 102/500 (20.4%) from blood and liquid culture.

For a better understanding, the results are presented in Venn diagrams, showing how many samples were amplified in each reaction and detected in more than one PCR (Fig 1).

Fig 1A shows the results of the PCR tests performed with the DNA extracted from whole blood samples. Nested and real-time PCR were the most sensitive tests for this type of sample, and only one sample had simultaneous detection in all four tests. Qualitative real-time PCR was the most efficient test for DNA extracted from liquid culture, showing amplification of 60 samples. Only four samples had simultaneous detection in all four PCR tests (Fig 1B). Fig 1C shows the results of all PCR tests performed with the DNA extracted from blood and liquid culture samples. The most efficient reaction to detect *Bartonella* spp. was real-time PCR with amplification of 72 samples, 45 of which were only in this test. Note that Fig 1C is not a product of the sum of the two previous Venn graphs. An example is that a sample positive only in ITS in blood was also positive when its liquid culture was tested in real-time PCR. This condition occurred in other samples as well.

To test the detection limit of each PCR, tests with known *Bartonella*-DNA concentrations were performed as previously described [36]. Although one genome equivalent (GE) of *B*. *henselae* was amplified in at least one of several reactions performed in each PCR technique (analytical sensitivity), the detection limit of each test, which refers to the minimum GE that amplified in all reactions (diagnostic sensitivity), was 50 GE in conventional PCR, 20 GE in real-time PCR and 10 GE in nested PCR. These data allow us to hypothetically calculate the amount of GE required in the initial sample for detection by the methods described in this study, considering 1) the detection limit of each reaction, 2) the amount of initial sample used in each diagnosis stage (extraction: 1 mL of liquid culture and 200 μL of blood/PCR: 5 μL in conventional and real-time PCRs and 2.5 μL in nested PCR), and 3) the dilution effect (in case of liquid culture) (Table 1).

**Table 1:** Initial amount of *Bartonella* sp.-DNA genome equivalent (GE) per mL of blood required in the initial sample for amplification.

	*Bartonella* sp. PCR		
Initial blood sample (GE/mL)	Conventional	Real-time	Nested
**Under 2,000**	-	-	-
**2,001 to 4,999**	-	+	+
**Over 5,000**	+	+	+

**Legend:** GE: Genome equivalent; (+): detection of *Bartonella* sp.-DNA; (-): no detection of *Bartonella* sp.-DNA.

### Sequencing

Seventy-two amplicons presented sufficient quality for Sanger sequencing, including the 11 ITS amplicons. It was possible to sequence samples from 66 of the 102 positive donors. In six donors, more than one region could be sequenced (Table 2). All samples presented 100% similarity to *B*. *henselae* (Access code in GenBank database for each region: *gltA* (KT945243.1- Bartonella henselae strain BR_LHR human 346 citrate synthase gene, partial cds), *ftsZ* (HG965802.1—Bartonella henselae strain BM1374163 complete genome, and ITS (BX897699.1-Bartonella henselae strain Houston1 complete genome).

**Table 2:** List of sequenced samples and regions.

Number of amplicons analyzed	Source	Sequenced region
11	Blood	*ftsZ*
2	Blood	*gltA*
3	Blood	ITS
1	Blood	ITS *& gltA*
19	Liquid culture	*ftsZ*
22	Liquid culture	*gltA*
3	Liquid culture	ITS
4	Liquid culture	ITS & *gltA*
1	Liquid culture	*ftsZ* & *gltA*

### Statistical analysis

**Fig 2:**
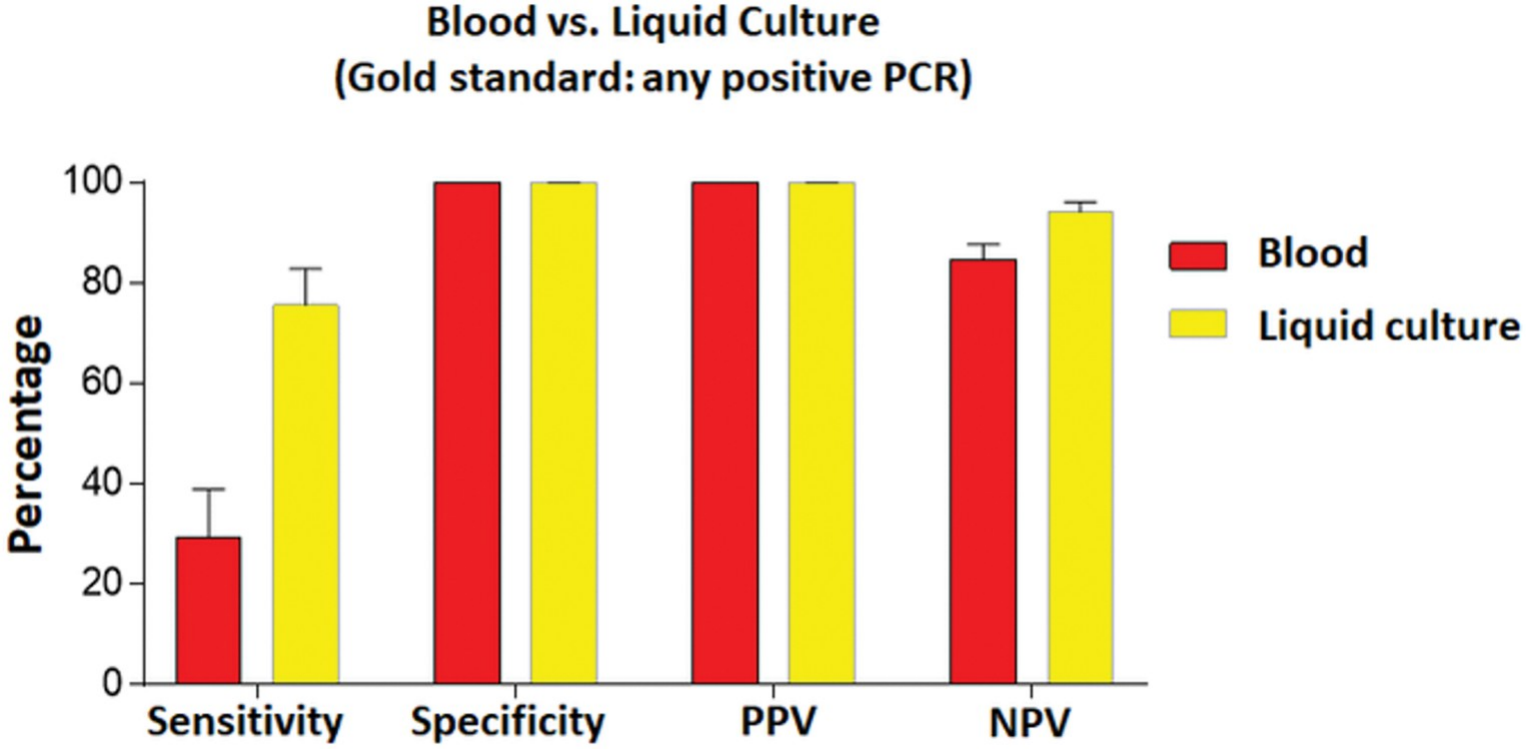
Comparison between results obtained analyzing blood versus liquid culture samples (considering ‘detectable in any PCR’ as the gold standard) by McNemar-Bowker test. PPV: positive predictive value; NPV: negative predictive value.

The McNemar-Bowker test was performed to determine the best sample (blood or liquid culture) to be used regardless of the PCR technique. The result of ‘detectable in any PCR’ was considered the gold standard. The data show that the reactions conducted in liquid culture were significantly more effective in detecting DNA of *Bartonella* spp. than those performed in blood (*p* < 0.0001). Liquid culture was also better in relation to the negative predictive value and the negative odds ratio (Fig 2 and Table B in S1 Appendix).

**Fig 3:**
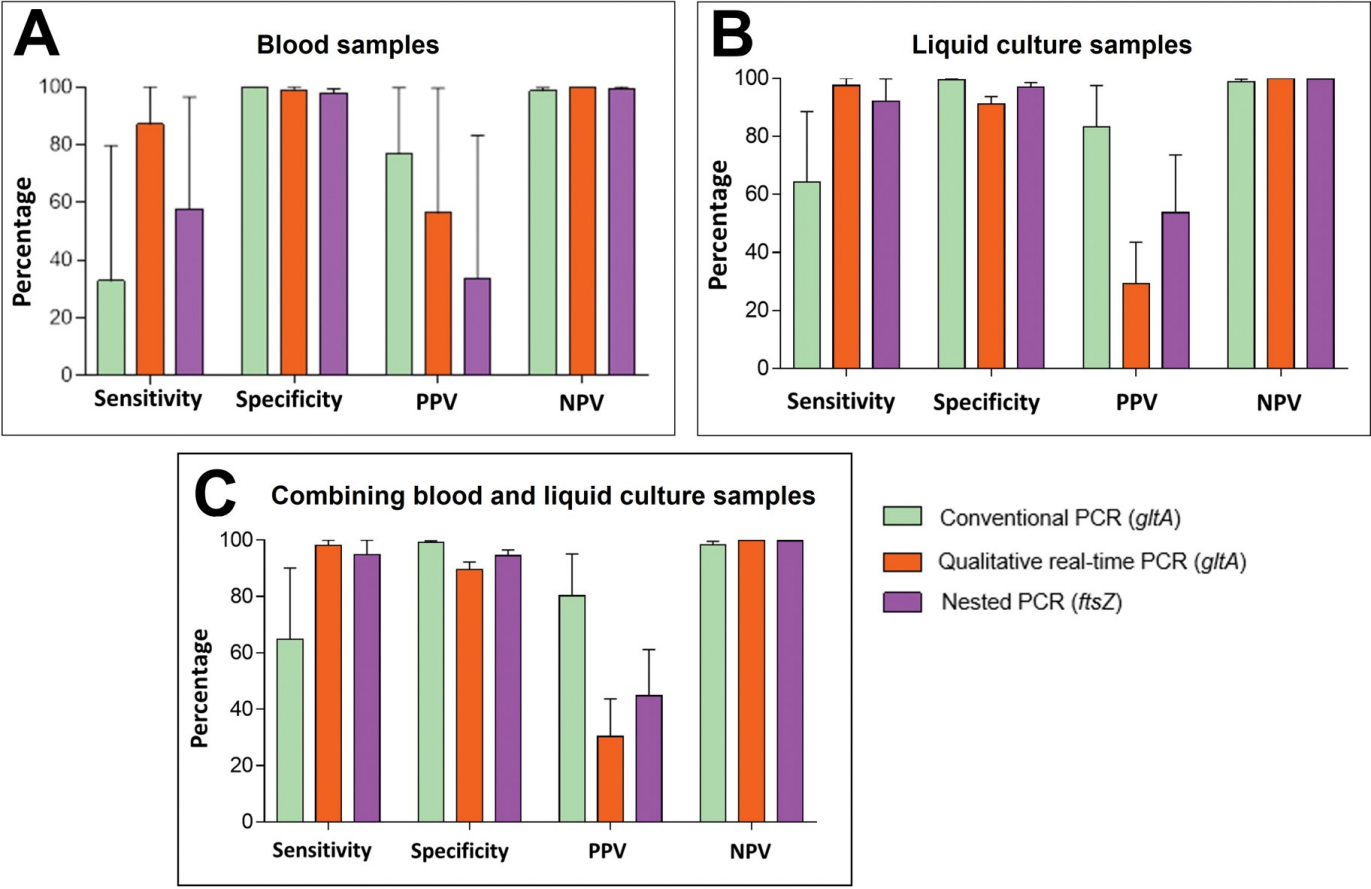
Comparison between different PCR tests for *B*. *henselae*. A: Blood samples; B: Liquid culture; C: Combining blood and liquid culture. PPV: positive predictive value; NPV: negative predictive value.

Bayesian LCM analysis was performed to determine the best PCR technique for blood or liquid culture samples and then to combine the results of blood and liquid culture, all of them using ‘detectable in any PCR’ as the gold standard (Fig 3). The results of conventional PCR for the ITS region were not used, as it was the only reaction targeting the *Bartonella* genus; all others targeted *B*. *henselae*.

**Fig 4:**
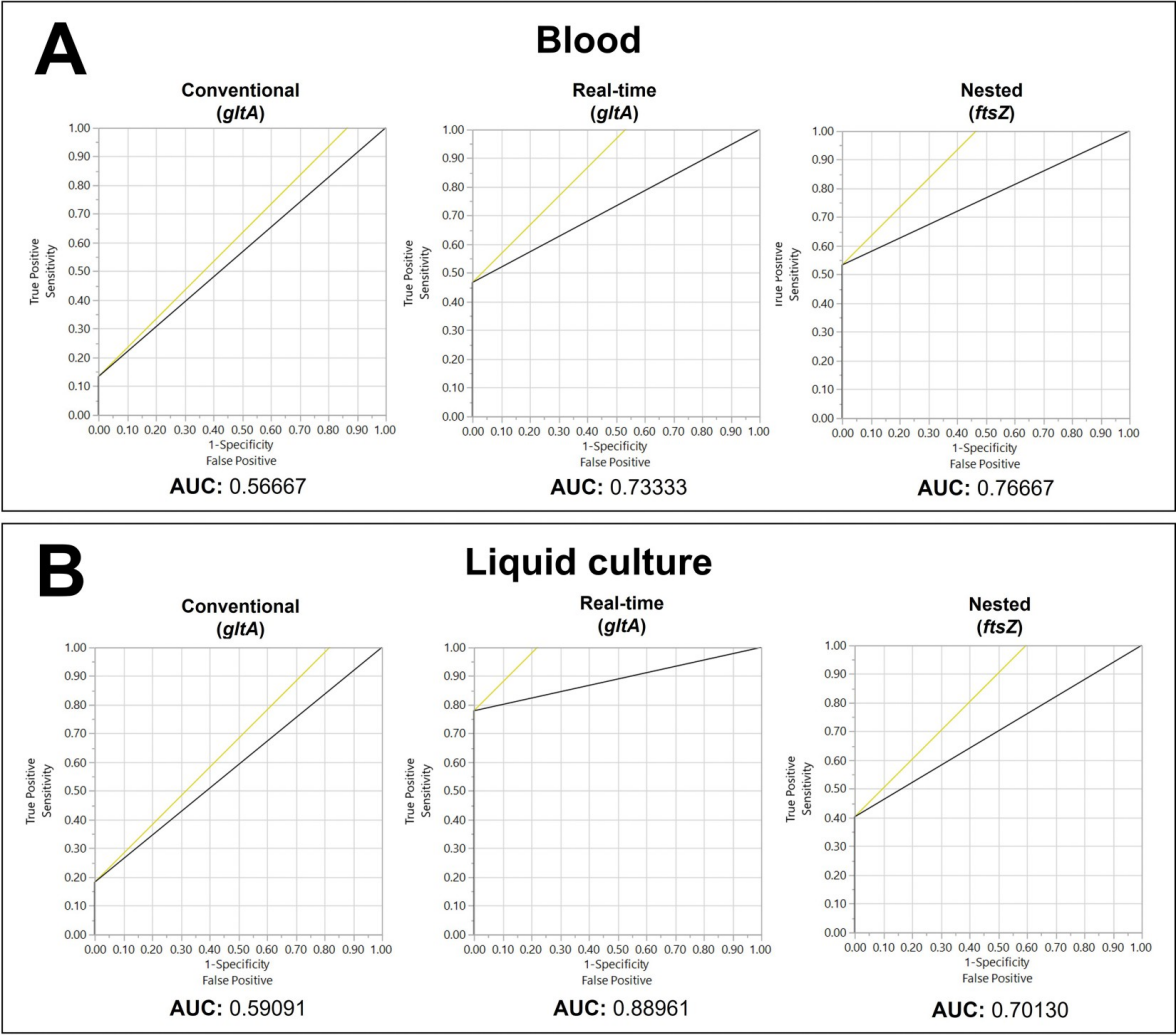
ROC (receiver operating characteristic) graphical representations generated from the results of different PCR tests for *Bartonella henselae* considering ‘detectable in any PCR’ as the gold standard. A: from blood samples; B: from liquid culture samples. AUC: Area under the curve. In the plot, the yellow 45-degree line marks the cutoff point that maximizes the sum of sensitivity and specificity.

Using the McNemar-Bowker test to analyze the results obtained only from whole blood samples, there was no difference in the concordance of results between nested PCR (*ftsZ*) and real-time PCR (*gltA*) (*p* value = 0.6547; McNemar-Bowker test, *p* > 0.05), demonstrating that they have similar diagnostic power in this type of sample (Fig 3A and Tables C and D in S1 Appendix). This result was confirmed by the ROC curve method, which showed very close AUC values for these PCRs (Fig 4A).

No agreement was found between the PCR results from liquid culture according to the McNemar-Bowker test, suggesting that sensitivity is different between them (Fig 3B and Tables E and F in S1 Appendix). ROC curve method analyses showed that the AUC of real-time PCR was greater than the AUC of other PCR tests, i.e., real-time PCR had greater discriminatory diagnostic power for liquid culture samples (Fig 4B).

Fig 3C shows the Bayesian LCM test combining liquid culture and blood sample PCR results, and together with the McNemar-Bowker test, it shows that the PCRs are different between them in this case (Tables G, H and I in S1 Appendix).

## Discussion

*B*. *henselae* was detected in 20.4% of donors (102/500) in our project, and comparing these data with the scientific literature, the prevalence found in our study is high. A survey about publications of prevalence of *Bartonella* sp. in blood donors published from 2009 to 2022 (Table J in S1 Appendix) have showed that only five from 20 studies used PCR as diagnostic method [37,47–65]. Two of these five were developed in Brazil, with prevalence from 1.8% to 3.2%, and in both only one screening PCR was performed with each sample [37,52].

According to our literature review, few studies have been conducted with humans using several combined molecular tests and many samples. We found only ten studies published during the same period (from 2009 to 2022) about *Bartonella* sp. prevalence using PCR that analyzed at least 30 individuals, excluding blood donors [66–74] (Table K in S1 Appendix). Few of them have used PCR for different regions. Therefore, data found in the literature are not sufficient to determine the exact prevalence of bacteremia caused by *Bartonella* spp. [75].

A study conducted in Spain by Portillo *et al*. [72] analyzed samples from 97 sanitary workers using several techniques, such as serology for different species, direct blood extraction, liquid and solid cultures, and different molecular tests of these cultures, such as conventional and real-time PCR, with different primers for the ITS region, in addition to sequencing. With this combination of diagnostic tests, they obtained 83.1% positive results in IFA tests, and the DNA of *Bartonella* spp. was amplified by 21.6%. This percentage of molecular detection in asymptomatic individuals is very similar to our data.

The present study again analyzed samples from 500 donors previously studied with just one PCR from liquid culture. The results obtained now from whole blood and liquid culture using different PCR methodologies were compared and showed that detection was lower in reactions that used DNA directly extracted from whole blood (6% or 30/500) compared to liquid culture (15.4% or 77/500). In the McNemar-Bowker test, liquid culture showed better sensitivity and negative predictive value when compared to whole blood, which can be explained by the fact that a diagnosis obtained directly from blood is less effective than after enrichment culture [76]. The samples are from asymptomatic blood donors, so they must have low-level bacteremia, i.e., they are below the detection level of diagnostic sensitivity. After enrichment culture, bacterial multiplication may occur to a number above this detection limit. In immunocompetent humans, bacteremia caused by *Bartonella* spp. is estimated to be 1–10 GE/μL (i.e., 1,000–10,000 GE/mL) [15], which may lead to the real possibility of false negatives. Liquid culture of *Bartonella* spp. increases the sensitivity of detection of infection caused by these bacteria by molecular methods [76–78].

Twenty-five samples had DNA from *Bartonella* spp. detected from whole blood but were undetected when tested in liquid culture. This fact can be explained by the ‘dilution effect’ [79]. There was no increase in the number of bacteria in the culture, but dilution occurred in a large volume of culture medium, so the concentration of bacteria in the liquid culture was below the detection level, indicating amplification of nonviable bacteria. The fastidious characteristic of the bacterium combined with the presence of growth inhibitors (such as the use of antimicrobials, even if occasionally used as anti-inflammatories, as seen with sulfone, hydroxychloroquine, etc.) make this hypothesis even more probable [35]. Currently, several techniques must be used concomitantly to avoid false negative results [66].

As with other diagnostic methods, there is no consensus on the best primers and conditions for PCR to detect the DNA of *Bartonella* spp.. Several studies describe the 16S rRNA gene region, the 16S-23S rRNA intergenic locus (ITS), the citrate synthase gene, or the riboflavin synthase gene, the *groEL* gene, the *ftsZ* gene, the *gltA* gene, or the RNA polymerase beta subunit as the most efficient and promising for the detection and differentiation of the various species of *Bartonella* [80,81]. In addition to the primers that determine the region to be amplified and therefore the sensitivity of the reaction, the PCR technique also influences the success of the diagnosis. Nested PCR and real-time PCR can greatly increase detection sensitivity [78,82–84]. In this study, the results obtained with statistical analysis show that conventional PCR in the *gltA* region is the least efficient technique to detect *Bartonella* sp.-DNA. Real-time PCR (*gltA*) is the best test for liquid culture, while for blood samples, no difference in sensitivity was found between nested (*ftsZ*) and real-time PCR (*gltA*) since confidence intervals for sensitivity overlap.

The great advantage of molecular diagnostic methods such as PCR is the fast result when compared to culture in addition to possible identification of the species causing the infection [85]. More sensitive and specific PCR tests allow quick diagnosis of the infection, even with low-level bacteremia. Maggi *et al*. developed, optimized, and validated droplet digital PCR (ddPCR), a new molecular technology, for the detection of *Bartonella* spp. DNA within several sample matrices. The ddPCR sensitivity (53/112) was significantly better than that of qPCR (6/112) when testing patient blood and enrichment blood culture samples [86]. Despite these advantages, PCR has some limitations: the possibility of false positive results due to contamination by control DNA or previously positive samples and false negative results for having less DNA than the detection limit. In addition, finding the pathogen DNA in the sample does not accurately indicate an active infection [79,87].

Several case reports [20,34,88–90] and a previous study with blood samples from cats [36] show that a combination of PCR and different samples increases the chances of detecting the pathogen. The results of this study agree with the literature and reinforce the need to combine several diagnostic tests to avoid false negatives.

In the previous project conducted with the same samples, the DNA of *Bartonella* spp. was detected in 16 donors [37]. Of these, the DNA was again amplified in only three samples, and only one that had been previously isolated by culture was detected again in real-time PCR. (Table L in S1 Appendix). This divergence in the results from the two studies developed with the samples of 500 blood donors shows how challenging the laboratory diagnosis of *Bartonella* spp. infection can be, since the samples from which isolates were previously obtained were supposed to have positive reactions, which did not happen in most samples. In the first study with these samples, only one conventional PCR genus-specific reaction was performed for the ITS region using DNA extracted from the liquid culture. Several factors can explain this divergence, including low-level bacteremia (1–10 GE/μL), especially for asymptomatic individuals. Therefore, there must be a small amount of pathogen DNA close to the detection limit. The aliquot used in the reaction may not have the amount required for amplification (Fig A in S1 Appendix).

Additionally, a stochastic (random) variation of the PCR amplification process occurs in the analysis of low amounts of DNA. Stochastic effects are seen as a fluctuation of results between replicated analyses [79]. For this reason, even a combination of several techniques does not prevent false negative results. In a previous study that used the same samples, the DNA of *Bartonella* spp. was detected in only 3.2% (16/500) of liquid culture samples using conventional PCR, and six of them were isolated in solid culture. In five of these six isolates, we were unable to detect *Bartonella* sp.-DNA [37].

Edouard *et al*. argue that to confirm a diagnosis of bartonelloses using exclusively the PCR technique, only samples with the DNA of *Bartonella* spp. detected in at least two different genome regions [68] should be considered. In this case, the possibility of false positives is reduced, and consequently, the specificity increases, but sensitivity is lost. Even considering this criterion, 35 (7%) samples were positive in our study with reactions in two different genome regions. If the six samples from which isolates were obtained in the previous study were added, we obtained 41 *B*. *henselae* DNA-detected samples (8.2%). None of the samples that originated these six isolates met the criterion of two distinct detected regions even in this current study using different PCRs.

As bartonelloses are caused by fastidious bacteria and low-level bacteremia is characteristic of the infection, it would not be advisable to use the criterion of two different gene region detections to confirm the diagnosis. Then, when adding up all the samples with *Bartonella* sp.-DNA detection in the two studies, 115/500 donors had *Bartonella* spp. detected, which corresponds to 23%. This result is close to the percentage of bloodstream infection in sanitary workers found in the study by Portillo *et al*. [72].

Cases reported in the literature [34,91,92] show that the low sensitivity of molecular tests may impact clinical practice. The five most common manifestations related to *Bartonella* spp. (CSD, bacillary angiomatosis, bacillary peliosis, culture-negative endocarditis and fever of undetermined origin) [93,94] are unquestionable and justify more investments in studies of this kind.

The laboratory diagnosis of *Bartonella* spp. is a challenge for several reasons: first, the fastidious characteristic of the bacterium, which makes laboratory culture an obstacle; second, the fact that it causes cyclic and low bacteremia; and finally, the lack of specific and sensitive tests for its diagnosis [15,95]. Combining methods is required to reduce false negatives. Further efforts should be dedicated to improving the diagnostic methods and ensuring better sensitivity to screen for infection by *Bartonella* spp..

The statistical analysis using all results of blood and liquid culture samples showed that, regardless of the sample, the sensitivity differs with the PCR types (conventional, nested and real-time PCR) and targets of gene regions.

The use of three different PCR tests with two types of samples (blood and liquid culture) increased the possibility of detecting *Bartonella* spp. considering that, in a previous project, 3.2% of blood donors were positive, and in this project, this rate increased to 20.4%. However, the combination of techniques did not prevent false-negative results since 13 donors who were positive in the previous project were not positive again.

## Conclusions

More than one-fifth of blood donors had at least one *B*. *henselae* DNA detected by a PCR test among the eight molecular reactions performed. Seven percent had the DNA detected for two or more distinct regions.

The statistical analysis using all results of blood and liquid culture samples showed that, regardless of the sample, the sensitivity differs with the PCR types (conventional, nested and real-time PCR) and targets of gene regions.

The results of our study indicate that public health authorities must review the risks and the impact of the transmission of *Bartonella* spp. through blood transfusions, especially for immunocompromised patients. Low-level bacteremia and the fastidious characteristics of the bacterium are challenges to laboratory diagnosis.

## Supporting information

S1 Appendix Table A: Primers used in this study. Table B. Statistical analysis (McNemar-Bowker test) of results from blood DNA samples versus liquid culture DNA samples (considering ‘detectable in any PCR’ as the gold standard). Table C. Bayesian latent class model (LCM) statistical analysis comparing the results of three distinct PCR tests for *B*. *henselae* from blood samples. Table D. Comparison of the agreement of results between each PCR technique performed with blood samples using the McNemar-Bowker test (*p* < 0.05). Table E. Bayesian latent class model (LCM) statistical analysis comparing the results of three distinct PCR tests for *B*. *henselae* from liquid culture samples. Table F. Comparison of the agreement of results between each PCR technique performed with liquid culture samples using the McNemar-Bowker test (*p* < 0.05). Table G. Bayesian latent class model (LCM) analysis comparing the results of three different PCRs for *B*. *henselae* performed with combined blood and liquid culture samples. Table H. Comparison of agreement of results between each PCR technique, regardless of liquid culture or blood sample, using the McNemar-Bowker test. Table I. Bayesian latent class model (LCM) analysis comparing the results of three different PCR tests for *B*. *henselae* regardless of liquid culture or blood sample, considering ‘detectable in any PCR’ as the gold standard. Table J. The Google Scholar, PubMed and Scopus databases were searched from 2009 to June 2022 for articles with ‘blood donors’ and ‘Bartonella’ in the title or abstract. NT: Not tested. Table K. The Google Scholar, PubMed and Scopus databases were searched from 2009 (year of sample collection for our study) to June 2022 for articles with ‘PCR’ and ‘Bartonella’ in the title or abstract (excluding studies that were not conducted with humans and that analyzed fewer than 30 individuals). Table L. Positive samples of PCR from a previous study with blood donors and results obtained in the current study. Fig A. Graphical representation of a variation in the amount of DNA in multiple PCRs.
(DOCX)
