# Supplementary material for: Comparison of molecular methods for Bartonella henselae detection in blood donors
Source: PLoS Negl Trop Dis. 2023 Jun 1;17(6):e0011336. doi: 10.1371/journal.pntd.0011336 (PMC10234562; doi:10.1371/journal.pntd.0011336)
Supplement: S1 Appendix — Table B. Statistical analysis (McNemar-Bowker test) of results from blood DNA samples versus liquid culture DNA samples (considering ‘detectable in any PCR’ as the gold standard). Table C. Bayesian latent class model (LCM) statistical analysis comparing the results of three distinct PCR tests for B. henselae from blood samples. Table D. Comparison of the agreement of results between each PCR technique performed with blood samples using the McNemar-Bowker test (p < 0.05). Table E. Bayesian latent class model (LCM) statistical analysis comparing the results of three distinct PCR tests for B. henselae from liquid culture samples. Table F. Comparison of the agreement of results between each PCR technique performed with liquid culture samples using the McNemar-Bowker test (p < 0.05). Table G. Bayesian latent class model (LCM) analysis comparing the results of three different PCRs for B. henselae performed with combined blood and liquid culture samples. Table H. Comparison of agreement of results between each PCR technique, regardless of liquid culture or blood sample, using the McNemar-Bowker test. Table I. Bayesian latent class model (LCM) analysis comparing the results of three different PCR tests for B. henselae regardless of liquid culture or blood sample, considering ‘detectable in any PCR’ as the gold standard. Table J. The Google Scholar, PubMed and Scopus databases were searched from 2009 to June 2022 for articles with ‘blood donors’ and ‘Bartonella’ in the title or abstract. NT: Not tested. Table K. The Google Scholar, PubMed and Scopus databases were searched from 2009 (year of sample collection for our study) to June 2022 for articles with ‘PCR’ and ‘Bartonella’ in the title or abstract (excluding studies that were not conducted with humans and that analyzed fewer than 30 individuals). Table L. Positive samples of PCR from a previous study with blood donors and results obtained in the current study. Fig A. Graphical representation of a variation in the amount [file pntd.0011336.s001.docx]

**PCR primers and conditions**

**Table A: Primers used in this study**

| **Reaction (gene)** | **Primer name** | **Primer** | **Reference** |
| --- | --- | --- | --- |
| Conventional (GAPDH) | GAPDH F | CCTTCATTGACCTCAACTACAT | Birkenheuer *et al.*[1] |
|  | GAPDH R | CCAAACTTGTCATGGATGACC |  |
| Conventional (ITS) | ITS F (325s) | CTTCAGATGATGATCCCAAGCCTTYTGGCG | Diniz *et al*.[2] |
|  | ITS R (1100as) | GAACCGACGACCCCCTGCTTGCAAAGCA |  |
| Conventional (*gltA*) | CS F | ATGGGTTTTGGTCATCGAGT | Staggemeier *et al*.[3] |
|  | CS R | AAATCGACATTAGGGTAAAGTTTTT |  |
| 1^st^ Reaction Nested (*ftsZ*) | BH F | GCCGCAAAGTTCTTTTCATG | Kawasato *et al*.[4] |
|  | BH R | AGGTGAACGCGCTTGTATTTG |  |
| 2^nd^ Reaction Nested (*ftsZ*) | BH S | CAAAACGGTTGGAGAGCAGT |  |
|  | BH A | CGCCTGTCATCTCATCAAGA |  |

***PCR conditions***

Conventional and nested PCRs were performed in a Thermocycler Techne 5000.

Real-time PCR was performed at StepOne Plus.

**GAPDH**

The conventional PCR targeting the GAPDH gene was performed on a 15 μL reaction mixture containing 0.15 μL of Taq DNA polymerase (GoTaq Flexi DNA Polymerase - Promega, 5U per μL), 0.6 μΜ of each primer, 0.6 μΜ of dNTP, 1.2 μL de MgCl_2_ (25mM) and 3 μL of DNA template. Amplifications were performed under the following conditions: one hot start cycle at 95⁰C for 2 minutes followed by 35 cycles of denaturing at 94⁰C for 15 seconds, annealing at 57⁰C for 15 seconds, and extension at 72⁰C for 15 seconds. Amplification was completed by an additional cycle at 72⁰C for 2 minutes, and products were analyzed by 1,5% agarose gel electrophoresis under UV exposure.

**ITS**

The conventional PCR targeting the 16S-23S rRNA gene intergenic transcribed spacer (ITS) of *Bartonella* species was performed on a 25 μL reaction mixture containing 0.125 μL of Taq DNA polymerase (GoTaq Flexi DNA Polymerase - Promega, 5U per μL), 0.5 μΜ of each primer (325s and 1100as), 0.5 μΜ of dNTP, 2 μL de MgCl_2_ (25mM) and 5 μL of DNA template. Amplifications were performed under the following conditions: one hot start cycle at 95⁰C for 5 minutes followed by 55 cycles of denaturing at 94⁰C for 15 seconds, annealing at 66⁰C for 15 seconds, and extension at 72⁰C for 15 seconds. Amplification was completed by an additional cycle at 72⁰C for 5 minutes, and products were analyzed by 1,5% agarose gel electrophoresis under UV exposure. A known concentration of *B. henselae* DNA was serially diluted 10-fold from 10^9^ to 1 genome equivalent (GE) per microliter to determine the sensitivity of the PCR assay. The sensitivity of this assay was established at a minimum of 50 genome equivalent (GE) of *B. henselae* per reaction tube.

**CS (*gltA)***

The conventional PCR targeting the gltA gene was performed on a 25 μL reaction mixture containing 0.125 μL of Taq DNA polymerase (GoTaq Flexi DNA Polymerase - Promega, 5U per μL), 0.4 μΜ of each primer (CS F and CS R), 0.5 μΜ of dNTP, 2 μL de MgCl_2_ (25mM) and 5 μL of DNA template. Amplifications were performed under the following conditions: one hot start cycle at 95⁰C for 5 minutes followed by 45 cycles of denaturing at 94⁰C for 30 seconds, annealing at 59⁰C for 30 seconds, and extension at 72⁰C for 30 seconds. Amplification was completed by an additional cycle at 72⁰C for 5 minutes, and products were analyzed by 1,5% agarose gel electrophoresis under UV exposure. A known concentration of *B. henselae* DNA was serially diluted 10-fold from 10^9^ to 1 genome equivalent (GE) per microliter to determine the sensitivity of the PCR assay. The sensitivity of this assay was established at a minimum of 50 genome equivalent (GE) of *B. henselae* per reaction tube.

**BH (*ftsZ*)**

**1^st^ PCR**

The first reaction of nested PCR targeting the *ftsZ* gene was performed on a 25 μL reaction mixture containing 0.125 μL of Taq DNA polymerase (GoTaq Flexi DNA Polymerase - Promega, 5U per μL), 0.4 μΜ of each primer (BH F and BH R), 0.5 μΜ of dNTP, 2 μL de MgCl_2_ (25mM) and 2,5 μL of DNA template. Amplifications were performed under the following conditions: one hot start cycle at 95⁰C for 5 minutes followed by 40 cycles of denaturing at 95⁰C for 30 seconds, annealing at 56⁰C for 30 seconds, and extension at 72⁰C for 30 seconds. Amplification was completed by an additional cycle at 72⁰C for 5 minutes, and products were analyzed by 1,5% agarose gel electrophoresis under UV exposure.

**2^nd^ PCR**

The second reaction of nested PCR targeting the *ftsZ* gene was performed on a 25 μL reaction mixture containing 0.125 μL of Taq DNA polymerase (GoTaq Flexi DNA Polymerase - Promega, 5U per μL), 0.4 μΜ of each primer (BH S and BH A), 0.5 μΜ of dNTP, 2 μL de MgCl_2_ (25mM) and 0.2 μL of first rection PCR product. Amplifications were performed under the following conditions: one hot-start cycle at 95⁰C for 5 minutes followed by 40 cycles of denaturing at 95⁰C for 30 seconds, annealing at 61⁰C for 30 seconds, and extension at 72⁰C for 30 seconds. Amplification was completed by an additional cycle at 72⁰C for 5 minutes, and products were analyzed by 1,5% agarose gel electrophoresis under UV exposure. A known concentration of *B. henselae* DNA was serially diluted 10-fold from 10^9^ to 1 genome equivalent (GE) per microliter to determine the sensitivity of the PCR assay. The sensitivity of this assay was established at a minimum of 10 genome equivalent (GE) of *B. henselae* per reaction tube.

**Qualitative Real-time PCR (*gltA*)**

The real-time PCR targeting the gltA of *Bartonella* *henselae* was performed on a 15μl reaction mixture contained 1X PCR mix (Fast SYBR Green Master Mix, Life Technologies), 0.5 pmol of each primer (CSF and CS R), and 5 μl of DNA template. Amplifications were performed under the following conditions: one hot-start cycle at 95⁰C for 20 seconds followed by 45 cycles of denaturing at 94⁰C for 3 seconds, annealing at 60⁰C for 30 seconds. After this, one hot start cycle at 95⁰C for 15 seconds, 60ºC for 60 seconds and an increases of 0.2 in 0.2°C up to 85°C to make melt curve. Amplification was detected by SYBR Green I fluorescence with emission at 522 nm. Melting curves.

Table B. Statistical analysis (McNemar-Bowker test) of results from blood DNA samples *versus* liquid culture DNA samples (considering ‘detectable in any PCR’ as the gold standard).

|  | **Blood** | **Liquid culture** |
| --- | --- | --- |
| **Sensitivity** | 29.4%  (CI: 21.4-38.9) | 75.5%  (CI: 66.3-82,8) |
| **Specificity** | 100.0%  (CI: 99.0-100.0) | 100.0%  (CI: 99.0-100.0) |
| **Positive predictive value** | 100.0%  (CI: 88.6-100.0) | 100.0%  (CI: 95.2-100.0) |
| **Negative predictive value** | 84.7%  (CI: 81.1-87.7) | 94.1%  (CI: 91.4-96.0) |
| **Positive odds ratio** | * | * |
| **Negative odds ratio** | 0.706  (CI: 0.623-0.800) | 0.245  (CI: 0.174-0.345) |

Legend: *Could not be calculated as it was 100% specificity; CI: confidence interval

Table C. Bayesian latent class model (LCM) statistical analysis comparing the results of three distinct PCR tests for *B. henselae* from blood samples.

|  | **Conventional PCR**  **(*gltA*)** | **Real-time PCR**  **(*gltA*)** | **Nested PCR**  **(*ftsZ*)** |
| --- | --- | --- | --- |
| **Sensitivity** | 32.8  (CI: 7.6-79.6) | 87.1  (CI: 34.1-100.0) | 57.8  (CI: 21.6-96.5) |
| **Specificity** | 99.8  (CI: 99.0-100.0) | 98.8  (CI: 97.2-100.0) | 97.8  (CI: 96.2-99.4) |
| **Positive predictive value** | 77.1  (CI: 27.5-99.8) | 56.3  (CI: 19.5-99.6) | 33.7  (CI: 11.3-83.2) |
| **Negative predictive value** | 98.7  (CI: 95.1-99.8) | 99.8  (CI: 96.7-100.0) | 99.2  (CI: 96.3-100.0) |

Legend: CI: confidence interval.

Table D. Comparison of the agreement of results between each PCR technique performed with blood samples, using the McNemar-Bowker test (*p* < 0.05).

| **Technique** | **Conventional PCR (*gltA*)** | **Nested PCR (*ftsZ*)** | **Real-time PCR (*gltA*)** |
| --- | --- | --- | --- |
| **Conventional PCR (*gltA*)** | - | 0.0027 | 0.0039 |
| **Nested PCR (*ftsZ*)** |  | - | 0.6547 |
| **Real-time PCR (*gltA*)** |  |  | - |

Table E. Bayesian latent class model (LCM) statistical analysis comparing the results of three distinct PCR tests for *B. henselae* from liquid culture samples.

|  | **Conventional PCR**  **(*gltA*)** | **Real-time PCR**  **(*gltA*)** | **Nested PCR**  **(*ftsZ*)** |
| --- | --- | --- | --- |
| **Sensitivity** | 64.2  (CI: 38.7-88.5) | 97.5  (CI: 76.2-100.0) | 92.1  (CI: 66.8-99.9) |
| **Specificity** | 99.5  (CI: 98.6-99.9) | 91.2  (CI: 88.2-93.7) | 97.0  (CI: 95.1-98.5) |
| **Positive predictive value** | 83.2  (CI: 58.0-97.5) | 29.3  (CI: 17.2-43.5) | 53.7  (CI: 33.0-73.6) |
| **Negative predictive value** | 98.7  (CI: 96.9-99.7) | 99.9  (CI: 98.8-100.0) | 99.7  (CI: 98.4-100.0) |

Legend: CI: confidence interval.

Table F. Comparison of the agreement of results between each PCR technique performed with liquid culture samples, using the McNemar-Bowker test (*p* < 0.05).

| **Technique** | **Conventional PCR (*gltA*)** | **Nested PCR (*ftsZ*)** | **Real-time PCR (*gltA*)** |
| --- | --- | --- | --- |
| **Conventional PCR (*gltA*)** | - | 0.0004 | <0.0001 |
| **Nested PCR (*ftsZ*)** |  | - | <0.0001 |
| **Real-time PCR (*gltA*)** |  |  | - |

**Table G.** Bayesian latent class model (LCM) analysis comparing the results of three different PCR reactions for *B. henselae* performed with combined blood and liquid culture samples.

|  | **Conventional PCR**  **(*gltA*)** | **Real-time PCR**  **(*gltA*)** | **Nested PCR**  **(*ftsZ*)** |
| --- | --- | --- | --- |
| **Sensitivity** | 64.8  (CI: 41.3-90.1) | 98.1  (CI: 81.9-100.0) | 94.8  (CI: 72.3-100.0) |
| **Specificity** | 99.3  (CI: 98.2-99.8) | 89.5  (CI: 86.4-92.3) | 94.5  (CI: 92.2-96.5) |
| **Positive predictive value** | 80.3  (CI: 57.0-95.1) | 30.4  (CI: 18.4-43.7) | 44.8  (CI: 27.5-61.2) |
| **Negative predictive value** | 98.4  (CI: 96.4-99.6) | 99.9  (CI: 98.9-100.0) | 99.8  (CI: 98.3-100.0) |

Legend: CI: confidence interval

**Table H.** Comparison of agreement of results between each PCR technique, regardless of liquid culture or blood sample, using the McNemar-Bowker test.

| **Technique** | **Conventional PCR (*gltA*)** | **Nested PCR (*ftsZ*)** | **Real-time PCR (*gltA*)** |
| --- | --- | --- | --- |
| **Conventional PCR (*gltA*)** | - | <0.0001 | <0.0001 |
| **Nested PCR (*ftsZ*)** |  | - | 0.003 |
| **Real-time PCR (*gltA*)** |  |  | - |

**Table I.** Bayesian latent class model (LCM) analysis comparing the results of three different PCR tests for *B. henselae* regardless of liquid culture or blood sample, considering **‘**detectable in any PCR’ as the gold standard.

|  | **Conventional PCR**  **(*gltA*)** | **Real-time PCR**  **(*gltA*)** | **Nested PCR**  **(*ftsZ*)** |
| --- | --- | --- | --- |
| **Sensitivity** | 17.6  (CI: 11.5-26.2) | 70.6  (CI: 61.1-78.6) | 46.1  (CI: 36.7-55.7) |
| **Specificity** | 100.0  (CI: 99.0-100.0) | 100.0  (CI: 99.0-100.0) | 100.0  (CI: 99.0-100.0) |
| **Positive predictive value** | 100.0  (CI: 82.4-100.0) | 100.0  (CI: 94.9-100.0) | 100.0  (CI: 92.4-100.0) |
| **Negative predictive value** | 82.6  (CI: 78.9-85.7) | 93.0  (CI: 90.2-95.0) | 87.9  (CI: 94.5-90.6) |

Legend: CI: confidence interval

**Table J.** Search in Scholar Google, Pubmed and Scopus databases from 2009 to June 2022 for articles with ‘blood donors’ and ‘Bartonella’ in the title or abstract.

| **Country** | **Positive PCR** | **Positive immunofluorescence test** | **Reference** |
| --- | --- | --- | --- |
| Turkey | NT | 48/800 (6%) IgG *B. henselae* | Yilmaz *et al.*  2009 [5] |
| Croatia | NT | 31/54 (57.4%) IgG *B. henselae* | Pandak *et al.*  2009 [6] |
| Greece | NT | 104/481 (21.6%) IgG *B. henselae* | Minadakis *et al.*  2009 [7] |
| Brazil | NT | 43/125 (34%) IgG *B. henselae* | Lamas *et al.*  2010 [8] |
| China | NT | 52/351 (14.81%) IgG *B. henselae* | Sun *et al.*  2010 [9] |
| Brazil | 1/56 (1.8%) | NT | Corrêa *et al.*  2012 [10] |
| Italy | NT | 13/122 (11.4%) IgG *B. henselae* | Mansueto *et al.*  2012 [11] |
| French Polynesia | NT | 0/472 (0%) IgG *B. henselae* | Musso *et al.*  2014 [12] |
| Namibia | NT | 3/319 (2.9%) IgG *B. henselae* | Noden *et al.*  2014 [13] |
| Turkey | NT | 11/333 (3.3%) IgG *B. henselae* | Aydin *et al.*  2014 [14] |
| Brazil | 16/500 (3.2%) | 81/500 (16.2%) IgG *B. henselae*   160/500 (32.0%) IgG *B. quintana* | Pitassi *et al.*  2014 [15] |
| Austria | NT | 1/100 (1%) IgG *B. henselae*  22/100 (22%) IgG *B. quintana* | Müller *et al.*  2016 [16] |
| Peru | 1/42 (2.38%) | NT | Pons *et al.*  2016 [17] |
| Korea | NT | 45/300 (15%) IgG *B. henselae* | Kwon *et al.*  2017 [18] |
| Chile | 19/140 (13.6%) | NT | Núñez *et al.*  2017 [19] |
| Poland | NT | 1/101(1%) IgG *B. henselae* | Brydak-Godowska *et al.*  2017 [20] |
| Poland | NT | 20/65 (30.8%) IgG *B. henselae*  2/65 (3.1%) IgM *B. henselae*  3/65 (4.6%) IgG *B. quintana*  1/65 (1.5%) IgM *B. quintana* | Łysakowska *et al*.  2019 [21] |
| Poland | NT | 9/199 (4.5%) IgG *B. henselae*  9/199 (4.5%) IgM *B. henselae* | Pawełczyk *et al.*  2019 [22] |
| Germany | NT | 0/96 (0%) IgG *B. bacilliformis* | Dichter *et al*.  2021 [23] |
| Poland | 1/65 (1.5%) | 18/65 (27.7%) IgG *Bartonella* sp.  2/65(3.1) IgM *Bartonella* sp. | Łysakowska *et al*.  2022 [24] |

NT: Not tested.

**Table K.** Search in Scholar Google, Pubmed and Scopus databases from 2009 (year of sample collection for our study) to June 2022 for articles with ‘PCR’ and ‘Bartonella’ in the title or abstract (excluding studies that were not conducted with humans and which analyzed less than 30 individuals).

| **Country** | **Number of patients/**  **population analyzed in study** | **Positive PCR**  **(sample)** | **Clinical manifestations** | **Reference** |
| --- | --- | --- | --- | --- |
| US | 192  patients exposed to animals | 46 (23.9%) *Bartonella* spp.  (blood, serum and liquid culture) | Fatigue, insomnia, joint pain and muscle pain | Maggi *et al.*  2011 [25] |
| US | 296  rheumatic patients | 40 (13.5%) *B. henselae* 54 (18.2%) *B. koehlerae* 10 (3.4%) *B. vinsonii subsp. berkhoffii*  (blood, serum and liquid culture) | Arthralgia/arthritis, Chronic fatigue and fibromyalgia | Maggi *et al.*  2012 [26] |
| France | 106  patients with endocarditis | 48/52 (92.0%) *Bartonella* spp.  (heart valves) 20/60 (33.0%) *Bartonella* spp.  (blood) 25/70 (36.0%) *Bartonella* spp.  (serum) | Endocarditis | Edouard *et al.*  2014 [27] |
| Spain | 89  veterinarians | 2 (2.25%) *B. henselae* 3 (3.37%) *B. vinsonii* subsp. *berkhoffii* 2 (2.25%) *B. quintana*  (blood and liquid culture) | Asymptomatic individuals | Oteo *et al.*  2017 [28] |
| France | 73  patients with CSD | 28 (38.36%) *Bartonella* spp.  (lymph node aspirate) | CSD (cat scratch disease) | Hobson *et al.*  2017 [29] |
| Poland | 51  patients with ocular inflammation | 0 (0%) *Bartonella* spp.  (intraocular lens fluid) | Ocular inflammation | Brydak-Godowska *et al.*  2017 [20] |
| India | 140  hospitalized patients | 3 (2.14%) *B. henselae*  (blood, lymph node aspirate and valve vegetation) | Fever and lymphadenopathy, culture-negative endocarditis and neuroretinitis. | Chaudhry *et al.*  2018 [30] |
| Spain | 97  sanitary workers | 21 (21.6%) *Bartonella* spp.  (blood, liquid culture and solid culture) | Asymptomatic individuals | Portillo *et al*.  2020 [31] |
| Brazil | 107  patients with CSD | 10/107(9.3%) *B. henselae*  (blood and liquid culture) | CSD (cat scratch disease) | Soares *et al.*  2020 [32] |
| Slovakia | 89 HIV-positive | 0 (0%) *Bartonella* spp. | HIV-positive patients | *Šimeková et al.*  2021 [33] |

The two studies by Maggi *et al.* [25, 26] were conducted with the same primers for conventional PCR (ITS region). Hobson *et al.* [29] also used conventional PCR only, but for the *ribC* region. Oteo *et al.* [28] used conventional and real-time PCR tests, but both with the same primers used in the studies by Maggi *et al.* [25, 26] (ITS). Edouard *et al.* [27] used PCR for different regions, but only in samples previously positive in real-time PCR for the ITS region, i.e., the other reactions for the *pap31* and *YOP* regions were used to confirm the first result. Brydak-Godowska *et al.* [20] tested the samples in conventional PCR for the *nuoG* region for *Bartonella* spp., but no sample was positive. Chaudhry *et al.* [30] tested different samples in conventional PCR for the citrate synthase (*gltA*) gene of *B. henselae*.

**Table L.** Positive samples of PCR from a previous study with blood donors and results obtained in the current study.

| **Donor** | **Positive sample (previous study)** | **PCR result in current study** | | | | |
| --- | --- | --- | --- | --- | --- | --- |
|  |  | **Conv.**  **ITS** | **Conv.**  **gltA** | **Nested** | **Real-time** |  |
| **108** | Liquid Culture/Isolate | - | - | - | - |  |
| **176** | Isolate | - | - | - | - |  |
| **234** | Isolate | - | - | - | - |  |
| **240** | Isolate | - | - | - | + Liquid Culture |  |
| **256** | Isolate | - | - | - | - |  |
| **487** | Isolate | - | - | - | - |  |
| **97** | Liquid Culture | - | - | - | - |  |
| **227** | Liquid Culture | - | - | - | - |  |
| **261** | Liquid Culture | - | + Liquid Culture | + Liquid Culture | + Liquid Culture |  |
| **271** | Liquid Culture | - | - | - | - |  |
| **294** | Liquid Culture | - | + Whole blood | + Whole blood | + Whole blood |  |
| **361** | Liquid Culture | - | - | - | - |  |
| **378** | Liquid Culture | - | - | - | - |  |
| **410** | Liquid Culture | - | - | - | - |  |
| **417** | Liquid Culture | - | - | - | - |  |
| **426** | Liquid Culture | - | - | - | - |  |

Legend: (+): detection of *Bartonella* sp.-DNA; (-): no detection of *Bartonella* sp.-DNA

**Figure**


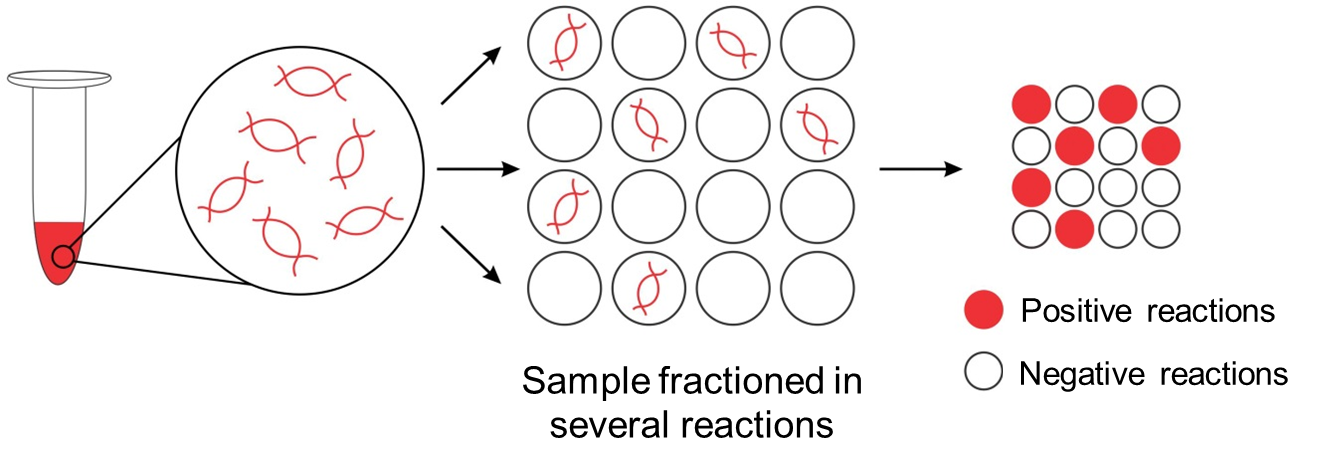


**Fig A.** Graphical representation of a variation in the amount of DNA in multiple PCR reactions

**References**

1. Birkenheuer AJ, Levy MG, Breitschwerdt EB. Development and evaluation of a seminested PCR for detection and differentiation of Babesia gibsoni (Asian genotype) and B. canis DNA in canine blood samples. J Clin Microbiol. 2003;41(9):4172-7. Epub 2003/09/06. PubMed PMID: 12958243; PubMed Central PMCID: PMCPMC193857.

2. Diniz PP, Maggi RG, Schwartz DS, Cadenas MB, Bradley JM, Hegarty B, et al. Canine bartonellosis: serological and molecular prevalence in Brazil and evidence of co-infection with Bartonella henselae and Bartonella vinsonii subsp. berkhoffii. Vet Res. 2007;38(5):697-710. doi: 10.1051/vetres:2007023. PubMed PMID: 17583666.

3. Staggemeier R, Pilger DA, Spilki FR, Cantarelli VV. MULTIPLEX SYBR® GREEN-REAL TIME PCR (qPCR) ASSAY FOR THE DETECTION AND DIFFERENTIATION OF Bartonella henselae AND Bartonella clarridgeiae IN CATS. Rev Inst Med Trop Sao Paulo. 2014;56(2):93-5. doi: 10.1590/S0036-46652014000200001. PubMed PMID: 24626408.

4. Kawasato KH, de Oliveira LC, Velho PE, Yamamoto L, Del Negro GM, Okay TS. Detection of Bartonella henselae DNA in clinical samples including peripheral blood of immune competent and immune compromised patients by three nested amplifications. Rev Inst Med Trop Sao Paulo. 2013;55(1):1-6. PubMed PMID: 23328718.

5. Yilmaz C, Ergin C, Kaleli I. [Investigation of Bartonella henselae seroprevalence and related risk factors in blood donors admitted to Pamukkale University Blood Center]. Mikrobiyol Bul. 2009;43(3):391-401. PubMed PMID: 19795614.

6. Pandak N, Daković-Rode O, Cabraja I, Kristof Z, Kotarac S. Prevalence of Bartonella henselae antibodies in children and blood donors in Croatia. Infection. 2009;37(2):166-7. doi: 10.1007/s15010-008-8113-0. PubMed PMID: 19274430.

7. Minadakis G, Chochlakis D, Kokkini S, Gikas A, Tselentis Y, Psaroulaki A. Seroprevalence of Bartonella henselae antibodies in blood donors in Crete. Scand J Infect Dis. 2008;40(10):846-7. doi: 10.1080/00365540802120000. PubMed PMID: 18609206.

8. Lamas CC, Mares-Guia MA, Rozental T, Moreira N, Favacho AR, Barreira J, et al. Bartonella spp. infection in HIV positive individuals, their pets and ectoparasites in Rio de Janeiro, Brazil: serological and molecular study. Acta Trop. 2010;115(1-2):137-41. doi: 10.1016/j.actatropica.2010.02.015. PubMed PMID: 20206113.

9. Sun J, Fu G, Lin J, Song X, Lu L, Liu Q. Seroprevalence of Bartonella in Eastern China and analysis of risk factors. BMC Infect Dis. 2010;10:121. doi: 10.1186/1471-2334-10-121. PubMed PMID: 20482887; PubMed Central PMCID: PMCPMC2886056.

10. Corrêa F, Pontes C, Verzola R, Mateos J, Velho P, Schijman A, et al. Association of Bartonella spp bacteremia with Chagas cardiomyopathy, endocarditis and arrhythmias in patients from South America. Braz J Med Biol Res. 2012;45(7):644-51. PubMed PMID: 22584639; PubMed Central PMCID: PMCPMC3854270.

11. Mansueto P, Pepe I, Cillari E, Arcoleo F, Micalizzi A, Bonura F, et al. Prevalence of antibodies anti-Bartonella henselae in western Sicily: children, blood donors, and cats. J Immunoassay Immunochem. 2012;33(1):18-25. doi: 10.1080/15321819.2011.591476. PubMed PMID: 22181817.

12. Musso D, Broult J, Parola P, Raoult D, Fournier PE. Absence of antibodies to Rickettsia spp., Bartonella spp., Ehrlichia spp. and Coxiella burnetii in Tahiti, French Polynesia. BMC Infect Dis. 2014;14:255. Epub 2014/06/03. doi: 10.1186/1471-2334-14-255. PubMed PMID: 24885466; PubMed Central PMCID: PMCPMC4022530.

13. Noden BH, Tshavuka FI, van der Colf BE, Chipare I, Wilkinson R. Exposure and risk factors to coxiella burnetii, spotted fever group and typhus group Rickettsiae, and Bartonella henselae among volunteer blood donors in Namibia. PLoS One. 2014;9(9):e108674. Epub 2014/09/27. doi: 10.1371/journal.pone.0108674. PubMed PMID: 25259959; PubMed Central PMCID: PMCPMC4178180.

14. Aydin N, Bulbul R, Telli M, Gultekin B. Seroprevalence of Bartonella henselae and Bartonella quintana in blood donors in Aydin province, Turkey. Mikrobiyol Bul. 2014;48(3):477-83. Epub 2014/07/24. PubMed PMID: 25052114.

15. Pitassi LH, de Paiva Diniz PP, Scorpio DG, Drummond MR, Lania BG, Barjas-Castro ML, et al. Bartonella spp. bacteremia in blood donors from Campinas, Brazil. PLoS Negl Trop Dis. 2015;9(1):e0003467. Epub 2015/01/16. doi: 10.1371/journal.pntd.0003467. PubMed PMID: 25590435; PubMed Central PMCID: PMCPmc4295888.

16. Muller A, Reiter M, Schotta AM, Stockinger H, Stanek G. Detection of Bartonella spp. in Ixodes ricinus ticks and Bartonella seroprevalence in human populations. Ticks Tick Borne Dis. 2016;7(5):763-7. Epub 2016/03/22. doi: 10.1016/j.ttbdis.2016.03.009. PubMed PMID: 26997137.

17. Pons MJ, Urteaga N, Alva-Urcia C, Lovato P, Silva J, Ruiz J, et al. Infectious agents, Leptospira spp. and Bartonella spp., in blood donors from Cajamarca, Peru. Blood Transfus. 2015;14(6):504-8. Epub 2015/12/18. doi: 10.2450/2015.0081-15. PubMed PMID: 26674831; PubMed Central PMCID: PMCPMC5111371.

18. Kwon HY, Im JH, Lee SM, Baek JH, Durey A, Park SG, et al. The seroprevalence of Bartonella henselae in healthy adults in Korea. Korean J Intern Med. 2017;32(3):530-5. doi: 10.3904/kjim.2016.010. PubMed PMID: 28490714.

19. Núñez M, Contreras K, Depix M, Geoffroy E, Villagra N, Mellado S, et al. Prevalence of Bartonella Henselae in Blood Donors and Risk of Blood Transmission in Chile. Revista chilena de infectologia : organo oficial de la Sociedad Chilena de Infectologia. 2017;34(6). doi: 10.4067/S0716-10182017000600539. PubMed PMID: 29488546.

20. Brydak-Godowska J, Kopacz D, Borkowski P, Fiecek B, Hevelke A, Rabczenko D, et al. Seroprevalence of Bartonella Species in Patients With Ocular Inflammation. Advances in experimental medicine and biology. 2017;1020. doi: 10.1007/5584_2017_19. PubMed PMID: 28405890.

21. Łysakowska M, Brzezińska O, Szybka M, Konieczka M, Moskwa S, Brauncajs M, et al. The Seroprevalence of Bartonella Spp. In the Blood of Patients With Musculoskeletal Complaints and Blood Donors, Poland: A Pilot Study. Clinical rheumatology. 2019;38(10). doi: 10.1007/s10067-019-04591-5. PubMed PMID: 31115789.

22. Pawełczyk A, Bednarska M, Kowalska J, Uszyńska-Kałuża B, Radkowski M, Welc-Falęciak R. Seroprevalence of Six Pathogens Transmitted by the Ixodes Ricinus Ticks in Asymptomatic Individuals With HIV Infection and in Blood Donors. Scientific reports. 2019;9(1). doi: 10.1038/s41598-019-38755-9. PubMed PMID: 30765826.

23. Dichter AA, Schultze TG, Wenigmann A, Ballhorn W, Latz A, Schlüfter E, et al. Identification of immunodominant Bartonella bacilliformis proteins: a combined in-silico and serology approach. Lancet Microbe. 2021;2(12):e685-e94. Epub 20210910. doi: 10.1016/S2666-5247(21)00184-1. PubMed PMID: 35544109.

24. Łysakowska ME, Szybka M, Olga B, Moskwa S, Konieczka M, Makowska J, et al. Cytokine and LL-37 gene expression levels in Bartonella spp. seropositive and seronegative patients of a rheumatology clinic. Adv Med Sci. 2022;67(1):163-9. Epub 20220310. doi: 10.1016/j.advms.2022.02.007. PubMed PMID: 35279619.

25. Maggi RG, Mascarelli PE, Pultorak EL, Hegarty BC, Bradley JM, Mozayeni BR, et al. Bartonella spp. bacteremia in high-risk immunocompetent patients. Diagn Microbiol Infect Dis. 2011;71(4):430-7. doi: 10.1016/j.diagmicrobio.2011.09.001. PubMed PMID: 21996096.

26. Maggi RG, Mozayeni BR, Pultorak EL, Hegarty BC, Bradley JM, Correa M, et al. Bartonella spp. bacteremia and rheumatic symptoms in patients from Lyme disease-endemic region. Emerg Infect Dis. 2012;18(5):783-91. doi: 10.3201/eid1805.111366. PubMed PMID: 22516098; PubMed Central PMCID: PMCPMC3358077.

27. Edouard S, Nabet C, Lepidi H, Fournier PE, Raoult D. Bartonella, a common cause of endocarditis: a report on 106 cases and review. J Clin Microbiol. 2015;53(3):824-9. doi: 10.1128/JCM.02827-14. PubMed PMID: 25540398; PubMed Central PMCID: PMCPMC4390654.

28. Oteo JA, Maggi R, Portillo A, Bradley J, García-Álvarez L, San-Martín M, et al. Prevalence of Bartonella spp. by culture, PCR and serology, in veterinary personnel from Spain. Parasit Vectors. 2017;10. doi: 10.1186/s13071-017-2483-z. PubMed PMID: 29116007.

29. Hobson C, Le Brun C, Beauruelle C, Maakaroun-Vermesse Z, Mereghetti L, Goudeau A, et al. Detection of Bartonella in cat scratch disease using a single-step PCR assay kit. J Med Microbiol. 2017;66(11):1596-601. Epub 2017/10/27. doi: 10.1099/jmm.0.000626. PubMed PMID: 29068281.

30. Chaudhry R, Kokkayil P, Ghosh A, Bahadur T, Kant K, Sagar T, et al. Bartonella henselae Infection in Diverse Clinical Conditions in a Tertiary Care Hospital in North India. The Indian journal of medical research. 2018;147(2). doi: 10.4103/ijmr.IJMR_1932_16. PubMed PMID: 29806608.

31. Portillo A, Maggi R, Oteo JA, Bradley J, García-Álvarez L, San-Martín M, et al. Bartonella Spp. Prevalence (Serology, Culture, and PCR) in Sanitary Workers in La Rioja Spain. Pathogens (Basel, Switzerland). 2020;9(3). doi: 10.3390/pathogens9030189. PubMed PMID: 32143533.

32. Soares TCB, Isaias GAB, Almeida AR, Drummond MR, da Silva MN, Lania BG, et al. Prevalence of Bartonella spp. Infection in Patients  with Sickle Cell Disease. Vector Borne Zoonotic Dis. 2020;20(7):509-12. Epub 20200203. doi: 10.1089/vbz.2019.2545. PubMed PMID: 32013778; PubMed Central PMCID: PMCPMC7336878.

33. Šimeková K, Soják Ľ, Víchová B, Balogová L, Jarošová J, Antolová D. Parasitic and Vector-Borne Infections in HIV-Positive Patients in Slovakia-Evidence of an Unexpectedly High Occurrence of Anaplasma phagocytophilum. Pathogens. 2021;10(12). Epub 20211129. doi: 10.3390/pathogens10121557. PubMed PMID: 34959511; PubMed Central PMCID: PMCPMC8704717.
